# The potential of 1.5 T magnetic resonance imaging for the evaluation of fetal anomalies of the great vessels

**DOI:** 10.3389/fped.2023.1136892

**Published:** 2023-03-28

**Authors:** Linjun Xie, Hong Xu, Xuelian He, Hang Fu, Lu Zhang, Wei Bai, Xuesheng Li, Li Bao, Huayan Xu, Xiaohong Li, Yingkun Guo

**Affiliations:** ^1^ Department of Radiology, Key Laboratory of Obstetric & Gynecologic and Pediatric Diseases and Birth Defects of Ministry of Education, West China Second University Hospital, Sichuan University, Chengdu, China; ^2^ Department of Ultrasound, Key Laboratory of Obstetric & Gynecologic and Pediatric Diseases and Birth Defects of Ministry of Education, West China Second University Hospital, Sichuan University, Chengdu, China; ^3^ Laboratory of Nervous System Injuries and Diseases, Center for Translational Medicine, Key Laboratory of Birth Defects and Related Diseases of Women and Children at Sichuan University, Ministry of Education, West China Second University Hospital, Sichuan University, Chengdu, China; ^4^ National Center for Birth Defects Monitoring of China, West China Second University Hospital, Sichuan University; Sichuan Birth Defects Clinical Research Center, West China Second University Hospital, Sichuan University, Chengdu, China

**Keywords:** fetal, magnetic resonance imaging, ultrasound, great-vessel anomalies, 1.5T

## Abstract

**Purpose:**

To determine the efficacy of 1.5 T magnetic resonance imaging (MRI) for the diagnosis of anomalies of the fetal great arteries with comparison to fetal ultrasound, and to compare image quality between 1.5 T and 3.0 T MRI in fetal imaging of the great arteries.

**Methods:**

We compared the results of postnatal exam or surgery and evaluated the application value of prenatal 1.5 T MRI in the assessment of fetal great-vessel anomalies. To further determine the diagnostic potential of 1.5 T MRI, 23 pregnant women with suspected fetal cardiovascular abnormalities who had undergone ultrasound and 3.0 T MRI were enrolled and compared, respectively.

**Results:**

Prenatal MRI was superior to ultrasound in demonstrating aortic arch and branch abnormalities (sensitivity, 92.86% vs. 83.33%; specificity, 66.67% vs. 20%). The mean quality ratings for fetal MRI at 1.5 T was higher than 3.0 T (*P* < 0.001). Other than the fast scan speed afforded by 3.0 T MRI, the signal noise ratio (SNR) of 1.5 T MRI were higher than those of 3.0 T MRI; however, the difference in contrast to noise ratio (CNR) between the two imaging modalities was not statistically significant.

**Conclusions:**

1.5 T MRI can achieve an overall assessment of fetal great-vessel anomalies, especially aortic arch and branch abnormalities. Therefore, 1.5 T MRI can be considered a supplementary imaging modality for the prenatal assessment of extracardiac great vessels malformations.

## Introduction

Among the congenital malformations, heart defects are the most common type of anomaly (1), affecting up to 9 per 1,000 live births (2). Great-vessel anomalies are significant parts of congenital heart disease. With the development of medical technology, the mortality associated with congenital heart defects is decreasing; however, it remains high compared with other birth defect (3). There are still difficulties to make the prenatal diagnosis of certain types of great vessels malformations. Moreover, the prognosis of some severe malformation is poor if not treated in time. Thus, early diagnosis of great-vessel anomalies can provide a basis for the prenatal management or postnatal treatment of these fetuses and reduce their postnatal mortality as well as improve the quality of life (4, 5).

Imaging plays a significant role in the prenatal diagnosis of congenital anomalies of the great vessels, especially ultrasound, which is the first-line choice in this setting. Although ultrasound has been the primary modality used in prenatal diagnosis, some conditions like obese mothers or poor fetal position may limit its utilization. Recently, the role of magnetic resonance imaging (MRI) in fetal diagnostic evaluation is increasing due to the high spatial resolution and objective image, especially in nervous system, assessment of growth and development or fetal heart (6). MRI technology has undergone a series of changes; fetal MRI technology has become an important means to prevent birth defects in our region. The American College of Radiology and Pediatric Radiology, and the Chinese Medical Association issued practice guidelines related to fetal MRI in 2015 and 2020, respectively (7, 8). Previous studies have demonstrated that 3.0 T MRI examination is safe for fetuses in the second and third trimesters and that 1.5 T is safe at any time during pregnancy (9). However, few studies have focused on the application of MRI with different field strengths to examine fetal great vessels. We followed up the fetuses who underwent great-vessel examination using 1.5 T fetal MRI to evaluate the value of this modality in diagnosing the associated great-vessel anomalies. Finally, our study tested and compared 1.5 T with 3.0 T MRI in fetal great-vessel assessment.

## Materials and methods

All studies were approved by the Ethics Committee of Sichuan University(K2019060). Informed consent was obtained from all participants prior to study participation. All participant-sensitive information was kept confidential and used solely for the purpose of this study.

### Patient population

We followed up pregnant women who underwent 1.5 T fetal MRI for great-vessel examination at our hospital. A total of 102 patients from our hospital were enrolled between April 2019 and August 2021. All of them underwent pregnancy ultrasound and 1.5 T MRI within one week. The follow-up included the results of amniocentesis, pregnancy outcomes, postnatal examinations, and surgical findings.

To compare the fetal great-vessel image quality between 1.5 T and 3.0 T MRI, we prospectively recruited pregnant women with suspected fetal great-vessel anomalies based on prenatal ultrasound by fetal cardiologist from October 2019 to July 2020. The study inclusion criteria were gestational age > 20 weeks, suspected fetal great-vessel anomalies on prenatal ultrasound, and no other contraindications to cardiac magnetic resonance (CMR). We excluded pregnant women who had undergone only one exam of the two MRI field strengths conducted (1.5 T or 3.0 T). Finally, 23 pregnant women out of the original 25 fulfilled these criteria and were included in the study.

### Ultrasound protocol

Fetal cardiac ultrasound examination is based on the guidelines of Chinese fetal cardiac ultrasound examination, including 11 standard sections, including transverse sternal section, four-chamber cardiac section, left ventricular outflow tract section, right ventricular outflow tract section, three-vessel section (3VV), three-vessel trachea section (3VT), long axis of aorta arch section, long axis of ductus arteriosus section, inferior vena cavo-right atrium section, short-axis of great vessels level section, short axis of left ventricle. Vanpraagh segmental analysis was used during the examination.

### MRI protocol

The MRI scanning position for pregnant women is mainly the supine feet-first position, supplemented by the lateral position. No sedative drugs were administered. After positioning image, a series of fetal transverse, coronal, and sagittal images were obtained from the thoracic inlet to below the diaphragm level. Scan specifications refer to the Chinese expert consensus on fetal MRI. In this study, no scans exceeded this recommended SAR. The specific parameters were as indicated in Table 1.

**Table 1:** Imaging parameters 1.5 T and 3.0T.

Parameters	1.5T	3.0T
Echo time(ms)	2.4 ms	2.0 ms
Repetition time(ms)	4.8 ms	5.5 ms
Thickness(mm)	4–6 mm	4–5 mm
FOV	300 × 252	320 × 289
Phase oversampling(%)	30%	30%
Voxel size(mm^3^)	1.2 × 1.2 mm	1.0 × 1.0 × 5 mm
Slice	15–35	15–35
Flip angle	70–75	58
Band width(Hz)	360–430	977
Acceleration(GRAPPA)	1.6–1.8	2
Scan duration(s)	20–50 s	20–30 s
Averages	1	1
Total scanning time	23.1 ± 8.9	35.4 ± 7.7*
Scanning effective time	9.6 ± 4.1	24.5 ± 8.1*
SAR	1.56 ± 0.17	1.62 ± 0.24

FOV, field of view; SAR, specific absorption ratio.

* *P *< 0.001.

The examinations were conducted in a 1.5 T MR scanner (Achieva dStream, Philips Healthcare, Best, Netherlands) equipped with a 16-channel body phased-array coil. The balanced steady-state free-precession sequence (BSSFP) was obtained using the following parameters: FOV, 300 × 252 mm; slice thickness, 4–6 mm with a 0-mm gap for short-axis stacks; TR/TE, 4.8 ms/2.4 ms; flip angle, 70°–75°; pixel bandwidth, 360–430 Hz; and acceleration factor (GRAPPA), 1.6–1.8.

In the 3.0 T MR scanner (MAGNETOM Skyra, Siemens Healthcare, Erlangen, Germany) equipped with an 18-channel body phased-array coil, the imaging parameters used to obtain the BSSFP (True FISP) sequences were as follows: FOV, 320 × 289-mm slice thickness, 4–5 mm with a 0-mm gap for short-axis stacks; TR/TE, 5.5 ms/2.0 ms; flip angle, 58°; pixel bandwidth, 977 Hz; and acceleration factor (GRAPPA), 2.

### Image analysis

All MRI data were analyzed using the commercially available software cvi42 (Circle Cardiovascular Imaging, Inc., Calgary, Canada). The great-vessel structures were evaluated by two experienced radiologists. If the results reported by them diverged, a consensus was established between the two radiologists. Both radiologists were blinded to the patients' ultrasound findings and the field strength. Using postnatal ultrasound, CT, MRI, or surgical findings as the reference standard, we compared the effectiveness of 1.5 T fetal MRI with that of ultrasound in identifying great-vessel anomalies.

Images were qualitatively examined by rating diagnostic image quality and the severity of motion artifacts. The 1.5 T and 3.0 T MRI images were scored on quality by an experienced radiologist. Quality scores were assigned based on the image quality using a 3-point rating system: 1, uninterpretable (anatomy inadequately defined and/or presence of severe artifact, rendering the image nondiagnostic); 2, suboptimal (well-defined anatomy over the majority of structures, with some artifacts at presentation; interpretation is possible but not ideal); and 3, good (well-defined anatomy over the entire great vessels, with no detected artifacts; comparable with postnatal MR imaging quality) (10). Image-artifact severity was based on the difficulty associated with the visualization of the underlying anatomy and was evaluated on a 4-point scale: 0, no artifact present; 1, mild artifact present (underlying anatomy well visualized); 2, moderate artifact present (underlying anatomy can be visualized, but delineation is suboptimal); and 3, severe artifact present (underlying anatomy cannot be visualized) (11). The signal intensity (SI) of the aorta, pulmonary artery, superior vena cava, and ventricular septum were measured. The standard deviation (SD) of the noise signal was measured from the ROI. The SNR and CNR values were calculated using the following formula: SNR = SI / SD and CNR = SNR1–SNR2. The CNR of the aorta–muscle, pulmonary artery–muscle, superior vena cava–muscle, and ventricular septal–muscle combinations were calculated by comparison with the chest-wall muscle.

### Statistical analysis

All statistical analyses were conducted using SPSS (version 24.0; IBM Corp., Armonk, NY, USA), MedCalc (version 15.8; MedCalc Software, Mariakerke, Belgium), and GraphPad Prism (version 7.0; GraphPad Software, San Diego, CA, USA). The sensitivity and specificity of the anomalies of the great vessels were calculated by comparing fetal MRI with ultrasound. Categorical variables are summarized as percentages, whereas *χ*^2^ or Fisher's exact tests were used to compare the categorical variables. Normal data were expressed as mean values with standard deviations. The homogeneity of the variance assumption was assessed using Levene's test. Significance was set to *P*-value < 0.05. The receiver operating characteristic (ROC) curves and areas under the ROC curve (AUCs) were used to compare the predictive performance of fetal MRI and ultrasound. We evaluated the intra and inter-observer agreement using weighted kappa coefficients, which were calculated by comparing the scores of the two readers and were interpreted as follows: <0.20, slight; 0.21–0.40, fair; 0.41–0.60, moderate; 0.61–0.80, substantial; and 0.81–1.0, almost perfect agreement. Significance was set to *P*-value < 0.05.

## Results

### Diagnostic value of 1.5 T MRI

**Figure 1:**
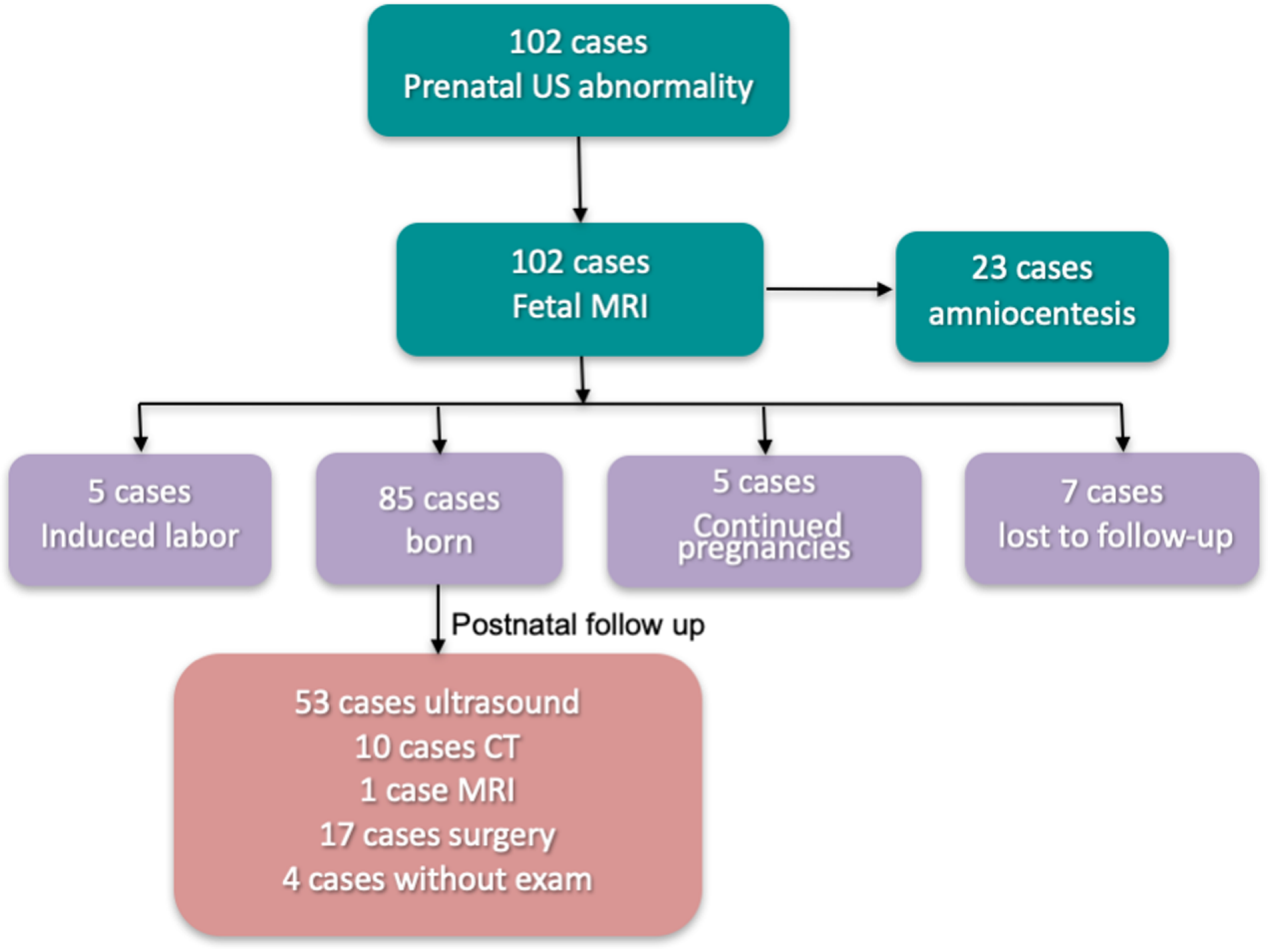
Flow chart of 102 cases of fetal follow-up.

**Figure 2:**
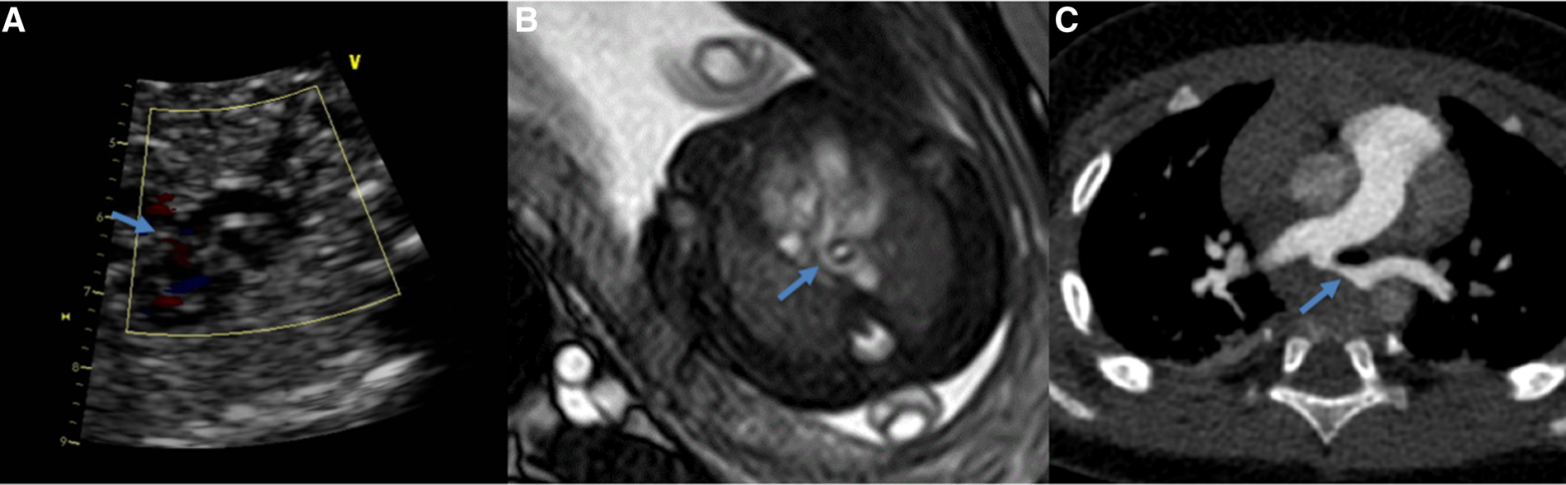
Comparing of the pulmonary artery sling obtained at ultrasound (**A**), 1.5T MRI (**B**) and postnatal CTA (**C**) (same fetus). The blue arrow show the left pulmonary artery.

**Figure 3:**
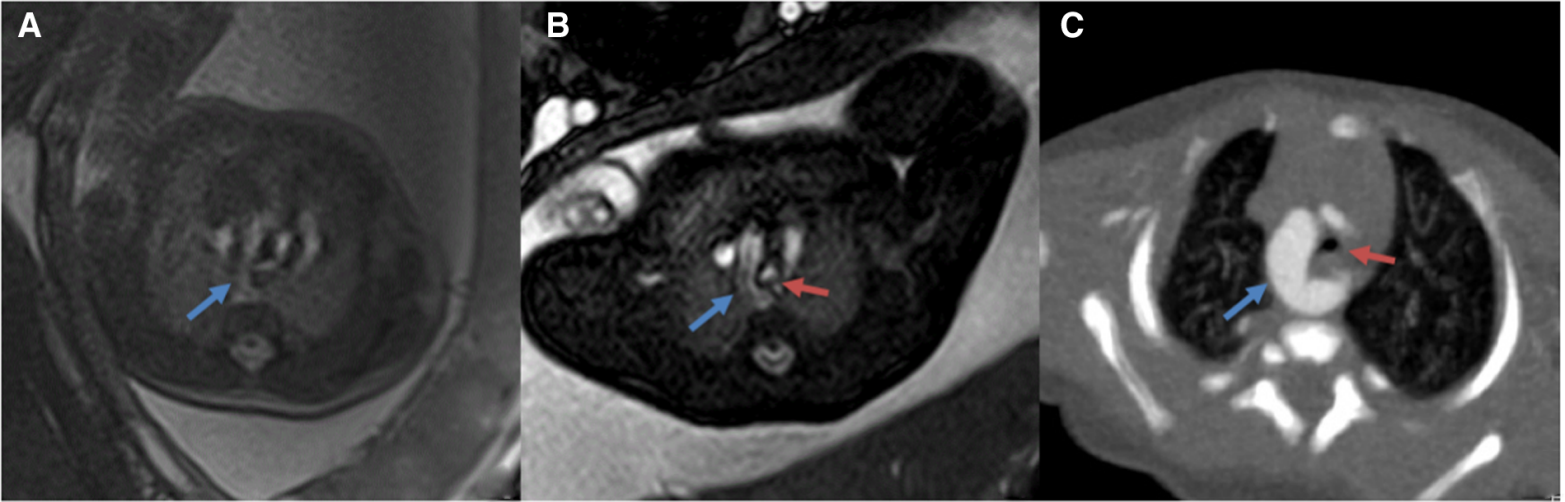
(**A,B**) comparison of 3.0 T(**A**) vs. corresponding 1.5 T (**B**) MR images of the fetal great vessels showing the advantages of increased resolution at 1.5 T (**C**): Postnatal CTA images (12 days) blue arrows show vascular ring; red arrows show the trachea.

We followed up pregnant women who underwent 1.5 T MRI fetal great-vessel examination at our hospital from April 2019 to August 2021. Of the 102 cases, 85 were born, five underwent induced labor, 5 were continued pregnancies, and seven were lost to follow-up (Figure 1). Among them, 23 cases underwent amniocentesis, and no obvious abnormality was observed. Of the 85 birth cases, 53 were followed up by ultrasound, 10 by CT, 1 by MRI, 17 by surgery, and 4 without exam. A total of 81 pregnant women were followed up by ultrasound, CT, MRI, or surgical treatment. The age range was 22–46 years, with an average age of 30.5 ± 4.5 years and a gestational age of 23–35 weeks at MRI. Figures 2, 3 present a comparison of the fetal MR images with postnatal CTA.

Aortic arch and branch abnormalities detected in this study include double aortic arch, coarctation of the aorta, right aortic arch, left subclavian artery, right subclavian artery, left aortic arch-right descending aorta, ascending aorta dilatation. Pulmonary vascular disease includes pulmonary artery sling, unilateral absence of pulmonary artery, pulmonary artery stenosis, crossed pulmonary arteries, anomalous pulmonary venous connection. Relative to the postnatal ultrasound, CT, MRI, or surgical findings, the results of 1.5 T fetal MRI and ultrasound are presented in Table 2. The sensitivity and specificity of these imaging modalities for diagnosing total great-vessel anomalies were 88.14% and 54.55% for fetal MRI and 86.44% and 13.64% for ultrasound, respectively; moreover, this difference was statistically significant (*P* = 0.017).

**Table 2:** A summary of the findings obtained with fetal MRI and ultrasound.

	Postnatal examination/surgical findings			
		+	−	Total
		59	22	81
Fetal MRI	+	52	10	62
	−	7	12	19
Fetal US	+	51	19	70
	−	8	3	11

**Figure 4:**
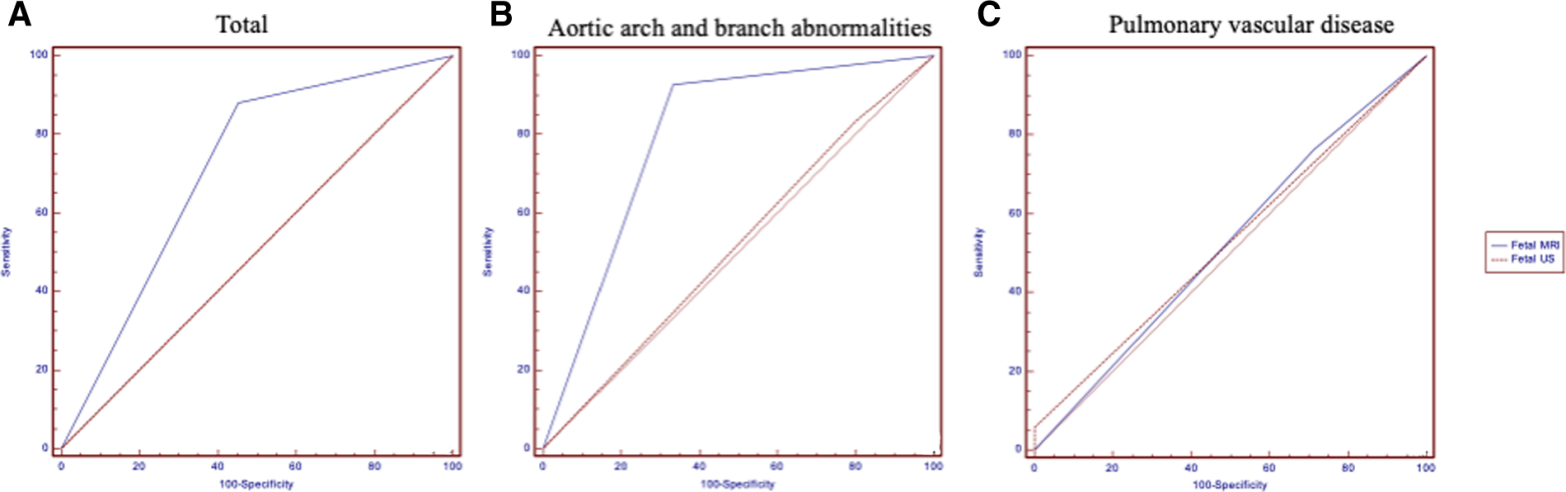
Comparing of the pulmonary artery sling obtained at ultrasound (**A**), 1.5 T MRI (**B**) and postnatal CTA(**C**) (same fetus). The blue arrow show the left pulmonary artery.

Table 3 shows the details of aortic arch and branch anomalies and pulmonary vascular disease. For fetal aortic arch and branch abnormalities, the sensitivity and specificity of fetal MRI were higher than those of ultrasound (sensitivity, 92.86% vs. 83.33%; specificity, 66.67% vs. 20%) (Table 4), with the difference being statistically significant (*P* = 0.038). Figure 4 presents the ROC curves and AUCs of fetal MRI and ultrasound. Fetal MRI (AUC = 0.798) exhibited a higher predictive ability than ultrasound in the diagnosis of aortic arch and branch abnormalities (AUC = 0.517). Fetal ultrasound missed aortic arch and branch abnormalities in seven cases (7/42), mainly involving the right aortic arch with the left subclavian artery (six cases). A total of 12 cases were misdiagnosed by fetal ultrasound (12/15), mainly involving coarctation of the aorta. Fetal MRI missed aortic arch and branch abnormalities in three case (3/42), involving the right aortic arch with the left subclavian artery. Five cases were misdiagnosed by fetal MRI (5/15), mainly involving coarctation of the aorta. The fetal MRI diagnosis of these 5 patients was false positive. Postnatal exam was confirmed to be normal.

**Table 3:** The diagnostic results of abnormalities detected by fetal MRI and ultrasound compared with follow up.

	Fetal MRI				Fetal US			
	TP	FN	TN	FP	TP	FN	TN	FP
**Aortic arch and branch abnormalities**								
Double aortic arch	13	0	0	0	13	0	0	0
CoA	10	0	10	5	10	0	3	12
RAA	8	0	0	0	8	0	0	0
RAA-ALSA	5	3	0	0	2	6	0	0
ARSA	1	0	0	0	1	0	0	0
Left aortic arch-right descending aorta	1	0	0	0	0	1	0	0
Ascending aorta dilatation	1	0	0	0	1	0	0	0
**Pulmonary vascular disease**								
Pulmonary artery sling	4	0	1	0	4	0	0	1
UAPA	0	2	0	0	2	0	0	0
Pulmonary artery stenosis	4	2	0	0	5	1	0	0
Crossed pulmonary arteries	2	0	1	4	2	0	0	5
Anomalous pulmonary venous connection	3	0	0	1	3	0	0	1

TP, true positive finding; FN, false negative finding; TN, true negative finding; FP, false positive finding; CoA, coarctation of the aorta; RAA, right aortic arch; ARSA, aberrant right subclavian artery; ALSA, aberrant left subclavian artery; UAPA, unilateral absence of pulmonary artery.

**Table 4:** Fetal aortic arch and branch abnormalities and pulmonary vascular disease obtained with fetal MRI and ultrasound.

	Aortic arch and branch abnormalities				Pulmonary vascular disease			
	MRI		US		MRI		US	
Postnatal examination/ surgical findings	+	−	+	−	+	−	+	−
+	39	3	35	7	13	4	16	1
−	5	10	12	3	5	2	7	0
Sensitivity (%)	92.86		83.33		76.47		94.12	
Accuracy (%)	85.96		66.67		62.6		66.67	

The sensitivity and specificity for the diagnosis of pulmonary vascular disease were 94.12% and 0% for ultrasound and 76.47% and 28.57% for fetal MRI, respectively. Fetal MRI and ultrasound exhibited no significant predictive ability in the context of the diagnosis of pulmonary vascular disease (*P* = 0.34). Fetal MRI missed pulmonary vascular disease in four cases (4/17), mainly involving the absence of a pulmonary artery, pulmonary stenosis, and a pulmonary artery sling. Five cases were misdiagnosed by fetal MRI (5/7), mainly involving crossed pulmonary arteries. Ultrasound missed pulmonary vascular disease in one case (1/17), which involved pulmonary artery sling. Seven cases were misdiagnosed by fetal ultrasound (7/7), mainly involving crossed pulmonary arteries.

### Comparison of 1.5 T vs 3.0 T MRI

#### Qualitative scoring of great-vessel images and artifact severity

A total of 23 pregnant women were enrolled in this study to compare the image quality between 1.5 T and 3.0 T MRI. The age range was 21–46 years, and the gestational age was 24–29 weeks. Suspected diseases included aortic coarctation, pulmonary artery stenosis, pulmonary artery sling, and anomalous pulmonary venous connection.

**Figure 5:**
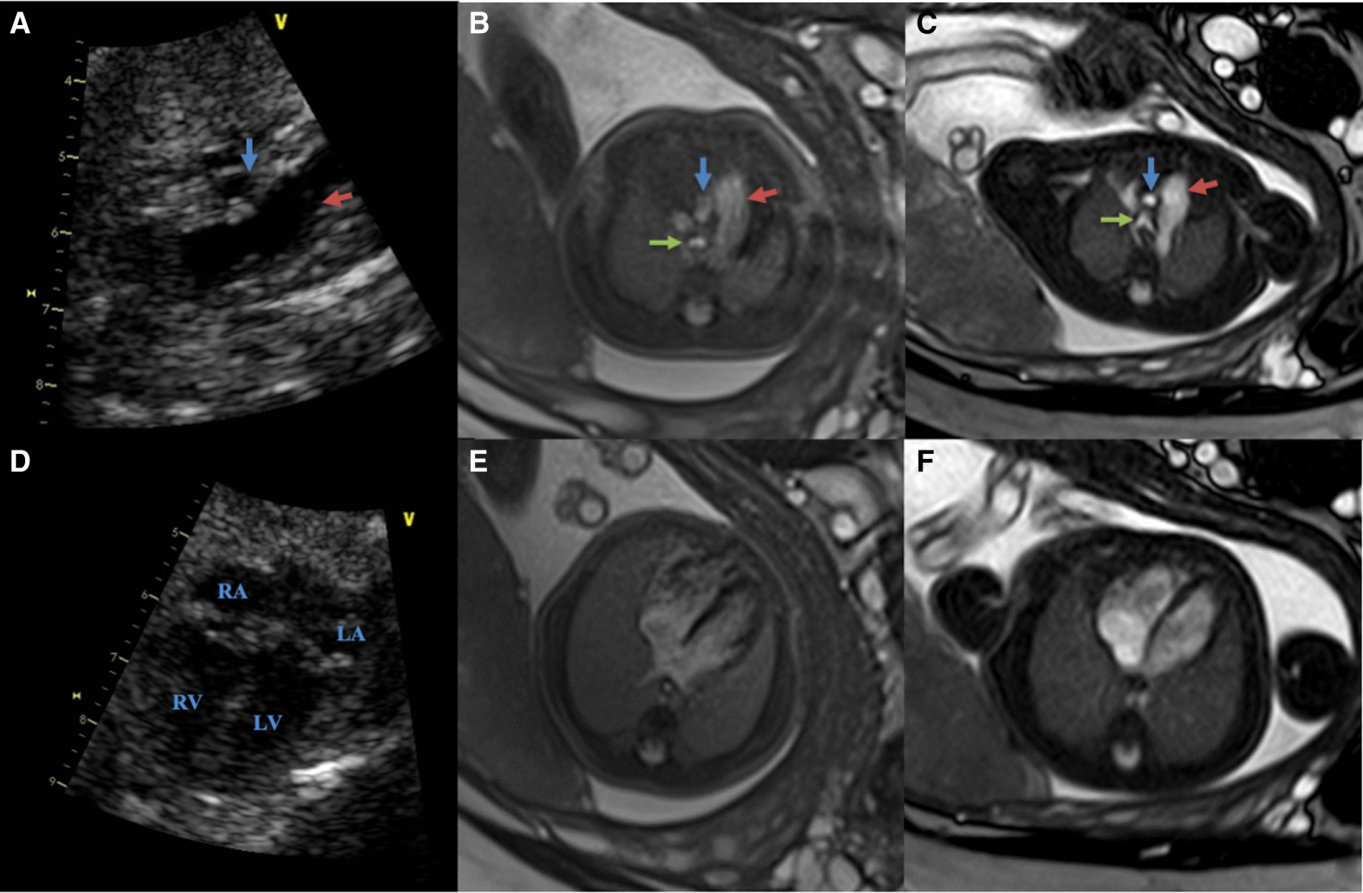
Comparing of the fetal cardiovascular at 26 wk 5 days gestation obtained at 3.0 T, 1.5 T MRI and ultrasound (same fetus) (**A–C**): ultrasound (**A**), 3.0 T (**B**) and 1.5 T(**C**) in the great vessels slice (red arrows show pulmonary artery; blue arrows show aorta; green arrows show the trachea) (**D–F**): ultrasound (**D**), 3.0 T (**E**) and 1.5 T(**F**) in the four-chamber slice both 1.5 T and 3.0 T scans were performed on the same day. The images from 1.5 T show superior tissue contrast and conspicuity to that of 3.0 T.

The fetal great-vessel MRI quality scores for 1.5 T and 3.0 T are presented in Table 5. Overall, 100% (23/23) of the patients who underwent 1.5 T MRI had an evaluable (Q2–3) image quality. In 3.0 T MRI, 35% (8/23) of the patients had an evaluable (Q2–3) image quality, whereas 65% (15/23) had a poor (Q1) image quality. The overall diagnostic quality ratings were higher (*P* < 0.001) for 1.5 T (mean, 2.30 ± 0.47) compared with 3.0 T MRI (mean, 1.35 ± 0.49) (Figure 5).

**Table 5:** Qualitative scoring of 1.5 T and 3.0 T images.

Frequency	1.5T	1, *n*	2, *n*	3, *n*	Total, *n* (%)
*3.0T*					
1, *n*		0	12	3	15 (65%)
2, *n*		0	4	4	8 (35%)
3, *n*		0	0	0	0 (0%)
Total, *n* (%)		0 (0%)	16 (70%)	7 (30%)	23

1, uninterpretable (anatomy inadequately defined and/or presence of severe artifact, rendering the image nondiagnostic); 2, suboptimal (well-defined anatomy over the majority, with some artifacts at presentation; interpretation is possible but not ideal); 3, good (well-defined anatomy over the entire great vessels, with no artifact present; comparable with postnatal MR imaging quality).

The image-artifact severity scores for the 1.5 T and 3.0 T groups are presented in Table 6. No artifact (Q0) was observed in 30% of patient studies using 1.5 T MRI, 48% had mild (Q1), 22% moderate (Q2) artifact and none had significant (Q3) artifact score. In contrast, 39% had moderate (Q2) and 61% severe (Q3) artifacts, whereas a Q0–1 score was not observed in the patient studies performed using 3.0 T MRI. The mean severity of the image artifacts was rated 2.61 ± 0.50 for 3.0 T MRI and 0.91 ± 0.73 for 1.5 T MRI (*P* < 0.001).

**Table 6:** Artifacts scoring of 1.5 T and 3.0 T images.

Frequency	*1* *.* *5T*	3, *n*	2, *n*	1, *n*	0, *n*	Total, *n* (%)
*3.0T*						
3, *n*		0	4	8	2	14 (61%)
2, *n*		0	1	3	5	9 (39%)
1, *n*		0	0	0	0	0 (0%)
0, *n*		0	0	0	0	0 (0%)
Total, *n* (%)		0 (0%)	5 (22%)	11 (48%)	7 (30%)	23

0, no artifact present; 1, mild artifact present (underlying anatomy well visualized); 2, moderate artifact present (underlying anatomy can be visualized but delineation is suboptimal); 3, severe artifact present (underlying anatomy cannot be visualized).

### Evaluation of the SNR and CNR

The SNR and CNR values of the images are presented in Table 7. The SNR of the aorta, pulmonary artery, and superior vena cava were higher on the 1.5 T images than on the 3.0 T images (*P* < 0.05). The SNR of the ventricular septum in 3.0 T images was higher than that in 1.5 T images (*P* = 0.002). The CNR of the aorta, pulmonary artery, superior vena cava, and ventricular septum in those two groups were not significantly different (all *P* *>* *0.05*).

**Table 7:** Comparison of SNR and CNR values for 1.5 T and 3.0T.

	1.5T	3.0T	*P*
SNR_aorta_	9.4 ± 3.8	6.0 ± 2.1*	0.001
SNR_pulmonary artery_	9.1 ± 3.0	5.8 ± 1.8*	<0.001
SNR_superior vena cava_	6.7 ± 3.4	4.6 ± 2.2*	0.027
SNR_ventricular septal_	4.7 ± 1.8	8.2 ± 3.9*	0.002
CNR_aorta−muscle_	4.2 ± 3.3	3.6 ± 2.5	0.389
CNR_pulmonary artery−muscle_	3.7 ± 3.3	3.5 ± 2.4	0.797
CNR_superior vena cava−muscle_	4.5 ± 4.1	4.0 ± 2.1	0.566
CNR_ventricular septal−muscle_	4.0 ± 3.5	3.7 ± 3.1	0.788

*P < 0.05 1.5T versus 3.0T.

#### Scan time

The total scanning time of 1.5 T MRI was less than that of 3 T (23.1 ± 8.9 min vs. 35.4 ± 7.7 min; *P* < 0.001). The effective time of 1.5 T MRI was less than that of 3 T (9.6 ± 4.1 min vs. 24.5 ± 8.1 min; *P* < 0.001). No significant difference was observed in the average SAR value between the two groups (1.56 ± 0.17 vs. 1.62 ± 0.24; *P *= 0.45).

In our study, 2 patients were jointly interpreted for 1.5 T quality scores assessment, 3 for 3.0 T quality scores, 2 for 1.5 T image-artifact severity scores and 2 for 3.0 T image-artifact severity scores. The kappa coefficient was measured between the intra and inter observer for the image quality evaluation. The kappa coefficients of the image quality score and image-artifact severity score were considered to have substantial and almost perfect agreement, respectively. The kappa coefficient agreements were 0.83, 0.67, 0.89 and 0.75(1.5 T quality scores, 3.0 T quality scores, 1.5 T image-artifact severity scores, 3.0 T image-artifact severity scores) for interobserver. The intraobserver kappa coefficient agreements were 0.76, 0.92, 0.94 and 0.91(1.5 T quality scores, 3.0 T quality scores, 1.5 T image-artifact severity scores, 3.0 T image-artifact severity scores).

## Discussion

With the development of treatment methods for cardiovascular diseases, some types of congenital heart diseases can be relieved or cured by perinatal surgery or elective surgical treatment, and early diagnosis can reduce perinatal morbidity and mortality. Ultrasound has a great advantage in the diagnosis of congenital intracardiac malformations; however, due to the limitations of acoustic window, maternal obesity, and amniotic fluid, there are certain difficulties in the diagnosis of extracardiac great-vessel malformation. Currently, there are few reports on the MRI-based diagnosis of fetal congenital heart disease. Manganaro et al. reported the MRI findings of suspected fetal congenital heart disease by ultrasound, indicating the possibility of fetal congenital heart disease diagnosis *via* MRI (12, 13). Ultrasound is used for fetal heart screening, and MRI is subsequently performed if the fetus is suspected of having abnormal great vessels.

Previous study reported that Fetal CMR diagnoses had been correct in 56.3% (14). The diagnostic accuracy of prenatal CMR was 95.6% in Li X's study (15). Taylor AM et al. previously published that overall sensitivity and specificity of fetal CMR was 72.7% and 96.2% respectively for detecting any cardiac pathology (16). Higher sensitivity of 92.6%, specificity of 99.1% were seen for major structural heart disease (14). In our study, the sensitivity, specificity and accuracy of fetal MRI for diagnosing total great-vessel anomalies were 88.14%, 54.55% and 79.0%. For fetal aortic arch and branch abnormalities, the sensitivity, specificity and accuracy of fetal MRI were 92.86%, 66.67% and 86.0% in our study. Compared with Taylor AM et al.'s study, we have similar sensitivity and the lower specificity. We analyze the reason of lower specificity was the misdiagnosis of the aortic coarctation by fetal MRI.

In our study, fetal MRI had a high sensitivity in detecting great-vessel anomalies (88.14% vs. 86.44%) compared with ultrasound, especially in the detection of aortic arch and branch abnormalities (92.86% vs. 83.33%). The explanations for the lower sensitivity levels observed for ultrasound in the detection of aortic arch and branch abnormalities might include the acoustic window and experience of the operator, especially when diagnosing the fetal great vessels in obese pregnant women. MRI has a certain value in the diagnosis of vascular rings, including the pulmonary sling, double aortic arch, right aortic arch, and left subclavian artery (17). Moreover, it has advantages over ultrasound in evaluating tracheal compression. Among the cases of a right aortic arch with left subclavian artery, six cases were considered to have a double aortic arch by fetal ultrasound. In addition, three cases were considered to have a double aortic arch detected by fetal MRI. Therefore, it is difficult to distinguish a double aortic arch from a right aortic arch with left subclavian artery based on fetal ultrasound and fetal MRI. This approach is mainly used for the differentiation of the left aortic arch and left vagal clavicular artery. In our study, fetal MRI missed one case of right coronary artery fistula, probably due to the heartbeat. Our study lacked ECG gating for fetal MRI imaging.

Five infants diagnosed with coarctation of the aorta on fetal MRI were normal after birth, and 10 infants diagnosed with coarctation of the aorta on fetal ultrasound were normal after birth. Therefore, fetal ultrasound and fetal MRI should be used with caution in the prenatal diagnosis of coarctation of the aorta. Accurate prediction of the fetuses that will develop coarctation of the aorta following birth is extremely challenging. Previous studies have concluded that the false-positive rate of ultrasound testing in this setting varies from 20% to 80% (18). The exact pathophysiological mechanisms leading to the development of coarctation of the aorta remain poorly understood. A possible explanation for this high false-positive rate is that the blood circulation during the fetal period is significantly different from the postnatal one. The most common site of aorta coarctation is the aortic isthmus. The blood flow of the fetal aortic arch mainly supplies the head, upper limbs, and coronary arteries, and only 10% of the blood supplies the descending aorta through the aortic isthmus; therefore, the coarctation of the aorta has a negligible effect on the hemodynamics of the fetus. After birth, the arterial ducts are closed, and the aortic isthmus stenosis is relieved. Lloyd et al. used 3D and phase-contrast MRI to evaluate suspected cases of fetal aorta coarctation; their multivariate logistic regression model correctly predicted the need for intervention in 93% of the cases (18).

In this study, ultrasound appeared to be superior to MRI in detecting pulmonary vascular anomalies. The specificity of ultrasound in the diagnosis of fetal pulmonary vascular disease was 0% in our study. A possible explanation for this finding is that ultrasound is the main imaging method used for prenatal diagnosis, and fetal MRI was performed only after suspected problems were reported. Pulmonary atresia was missed in two cases by fetal MRI. The missed diagnosis of pulmonary artery atresia on one side by fetal MRI may be related to the poor display of the pulmonary bifurcation at the MRI scan level. Four of the fetuses diagnosed with crossed pulmonary arteries by MRI and five cases diagnosed by ultrasound had normal pulmonary arteries following birth. Pulmonary artery bifurcation is difficult to clearly visualize on fetal MRI slices. We recommend scanning at a negative interval and oblique angles.

This study evaluated the primary diagnosis of great vessels and did not compare the diagnosis of fetal intracardiac structures. In the evaluation of atrial defects, ventricular defects, and valvular regurgitation, ultrasound has the advantage of using Doppler technology (19, 20). Therefore, ultrasound is the first choice for fetal heart structure examination. Fetal heart screening is usually performed in the second trimester. For the detection of great-vessel abnormalities, if the ultrasound diagnosis is doubtful, MRI can be used for further diagnosis. In particular, the risk stratification and delivery plan of the fetus following birth, including the decision of Cesarean section or vaginal delivery and whether assisted ventilation is immediately required after birth will provided. Consultations for parents on diagnosis, postpartum outcomes, possible surgical methods, and postpartum care should be provided.

Generally, the gestational age for fetal MRI great-vessel examination is relatively low, and the cardiac and vessel pulsation artifacts cause certain difficulties in this diagnosis. High field strengths have better tissue resolution; however, they are associated with a more frequent occurrence of artifacts (21). Therefore, the two field strengths should be compared to determine which one is more useful for fetal imaging. At present, no studies have evaluated fetal great-vessel imaging using different magnetic resonance field strengths. Our study evaluated 1.5 T and 3.0 T fetal MRI great-vessel examination regarding the scan time, SNR/CNR, image quality score, and artifact severity score.

The SNR values of the aorta, pulmonary artery, and superior vena cava at 1.5 T were higher than those obtained at 3.0 T MRI, indicating that 1.5 T MRI yields better visualization of the structures of the aorta, pulmonary artery, and superior vena cava compared with 3.0 T MRI. Regarding the SNR values of the ventricular septum, 3.0 T was better than 1.5 T MRI. Because the relative position of the ventricular septum is fixed, it is less affected by the pulsation of the aorta, and the tissue resolution of 3.0 T is higher than that of 1.5 T MRI. The CNR difference between 1.5 T and 3.0 T MRI was not statistically significant, suggesting that there is no difference between these modalities in the ability to distinguish different tissues. The image quality score of 1.5 T MRI, which was 100%, met the diagnostic requirements, i.e., that 30% of the images exhibit a good score, whereas only 35% of the 3.0 T MRI images met the diagnostic requirements. In terms of meeting the clinical diagnosis, 1.5 T was far better than 3.0 T MRI. The artifact severity score of 3.0 T was significantly higher than that of 1.5 T MRI, indicating that the 3.0 T images were significantly affected by artifacts. A possible explanation for this observation is that a higher magnetic field strength yields more obvious motion artifacts. In the analysis of the total scanning time and effective scanning time, 1.5 T was superior to 3.0 T MRI.

### Limitations

The present study had several limitations. First, fast fetal heart rates and the lack of ECG-gating induced more artifacts, which may require faster acquisition speed. Fetal MRI technology needs to be improved in future research. Second, only pregnant women with abnormal fetal ultrasound or suspected abnormalities will have fetal MRI exam. Part of the observed result may be due to referral bias. Third, not all follow-up patients were reviewed using imaging methods.

## Conclusion

Fetal MRI can be employed to examine fetal great-vessel anomalies, including associated malformations, especially in the aorta and its branches. In summary, regarding scan time, image quality, and artifact severity score, 1.5 T MRI was superior to 3.0 T MRI. Therefore, fetal MRI can be considered as a supplementary imaging modality for prenatal diagnosis.

## Data Availability

The raw data supporting the conclusions of this article will be made available by the authors, without undue reservation.
